# Minimally Invasive Approach to Eliminate Pyogenic Granuloma: A Case Report

**DOI:** 10.1155/2012/909780

**Published:** 2012-01-26

**Authors:** B. Chandrashekar

**Affiliations:** Department of Periodontics, K.B.H. Dental College and Hospital, Nashik, Maharashtra, India

## Abstract

Pyogenic granuloma is one of the inflammatory hyperplasia seen in the oral cavity. The term is a misnomer because it is not related to infection and arises in response to various stimuli such as low-grade local irritation, traumatic injury, or hormonal factors. It is most commonly seen in females in their second decade of life due to vascular effects of hormones. Although excisional surgery is the treatment of choice for it, this paper presents the safest and most minimally invasive procedure for the regression of pyogenic granuloma.

## 1. Introduction

Pyogenic granuloma is a hyperactive benign inflammatory lesion that occurs mostly on the mucosa of females with high levels of steroid hormones. It is generally believed that female sex hormones play important roles in its pathogenesis [1, 2]. It is a tumourlike growth of the oral cavity frequently located surrounding the anterior teeth or skin that is considered to be neoplastic in nature [3, 4]. It usually arises in response to various stimuli such as low-grade local irritation [3, 5], traumatic injury, hormonal factors [6], or certain kinds of drugs [7].

 The term “pyogenic granuloma” is a misnomer because the lesion does not contain pus and is not strictly speaking a granuloma [1, 3, 5, 8]. Approximately one-third of the lesions occur due to trauma [9], and poor oral hygiene may also be one of the precipitating factors [1, 3, 8]. It often presents as a painless, pedunculated, or sessile mass of gingiva. 

## 2. Clinical Presentation

**Figure 1:**
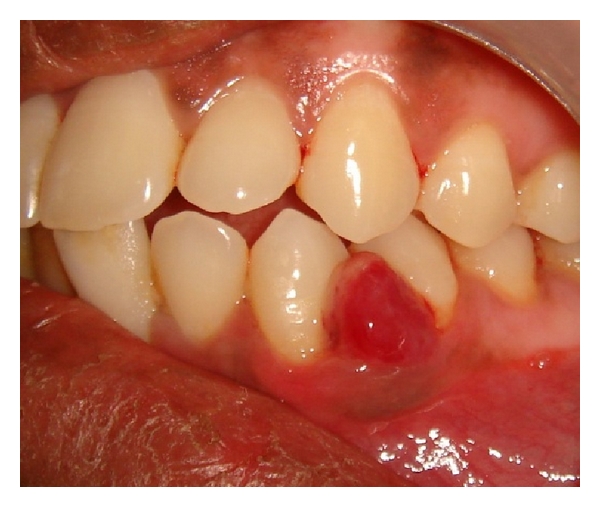
Exophytic and hemorrhagic lesion in the lower canine-premolar region (Buccal view).

**Figure 2:**
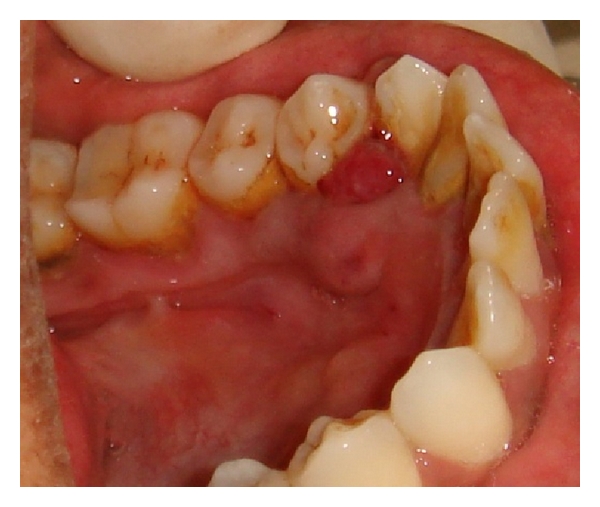
Exophytic and hemorrhagic lesion in the lower canine-premolar region (lingual view).

A female patient of 28 years old came to our outpatient department with a chief complaint of bleeding gums particularly in the lower left premolar region while brushing and chewing food. She also gave a history of asymptomatic soft tissue growth in the mandibular premolar area which was increasing in size gradually since 4 weeks. Patient's medical history was noncontributory, and on clinical examination there was a smooth exophytic lesion manifested as a small erythematous papule on a pedunculated base which is hemorrhagic with spontaneous bleeding on probing the area with soft tissue growth (Figures 1 and 2). The lesion was painless and asymptomatic except for the slight discomfort to the patient due to the growth. Physical examination revealed no other abnormalities, and there was no cervical lymphadenopathy. On hard tissue examination there were 28 teeth with atraumatic occlusion and crowding of lower anteriors. There was moderate supra- and subgingival calculus with moderate gingivitis.

### 2.1. Diagnosis

**Figure 3:**
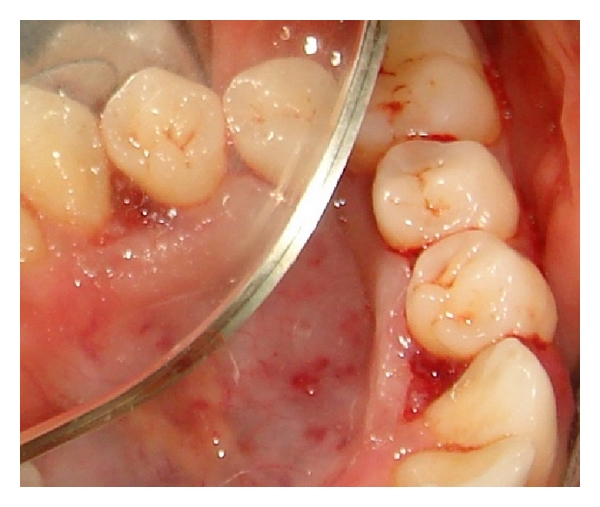
Incisional biopsy done on the lingual side.

So by considering all the above features a provisional diagnosis was made as pyogenic granuloma and incisional biopsy was performed under local anaesthesia and sent for histological examination (Figure 3). Based on histological report it was finally diagnosed as pyogenic granuloma.

### 2.2. Management

The treatment decided was complete scaling and curettage especially in the premolar area where there was soft tissue growth. There was severe bleeding while doing scaling and curettage. However, bleeding stopped within few minutes by applying pressure with gauze. The patient was advised to perform and maintain thorough oral hygiene by brushing twice a day and to use chlorhexidine mouth rinse of 0.12% twice daily.

**Figure 4:**
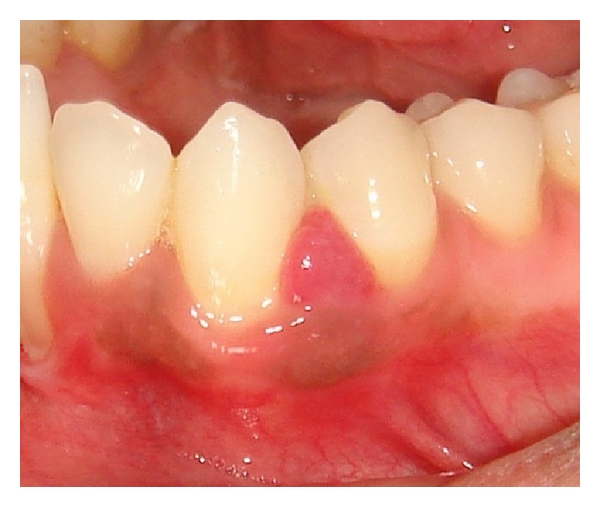
Size of the lesion after 7 days of nonsurgical treatment.

**Figure 5:**
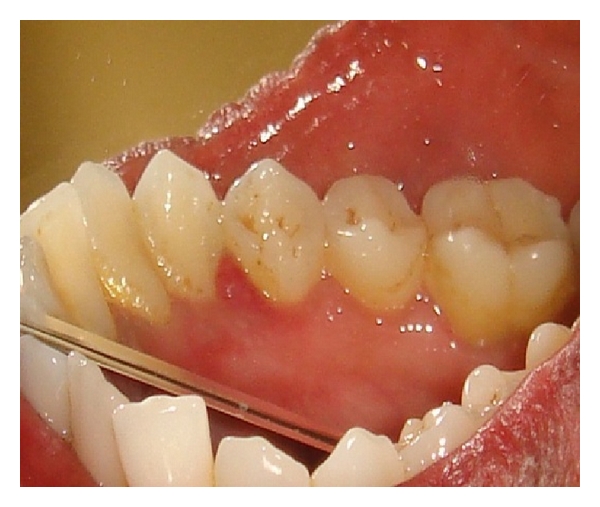
Size of the lesion after 4 weeks of nonsurgical treatment.

**Figure 6:**
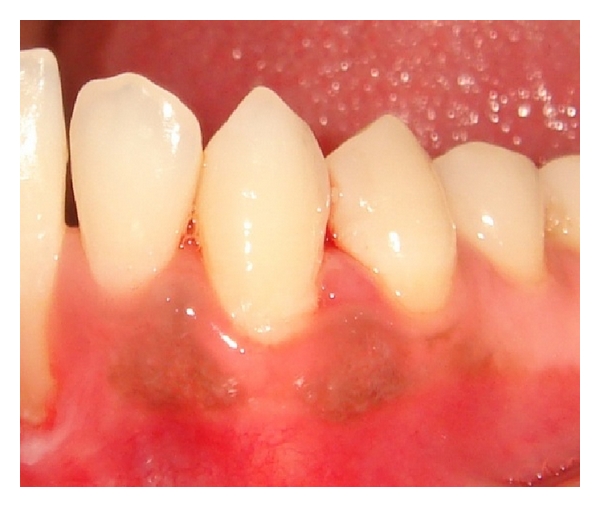
Size of the lesion after 6 months of nonsurgical treatment.

On observation, there was gradual reduction in the growth after the first week (Figure 4). So, it was further decided to treat the lesion in noninvasive approach, and later, every week, a thorough scaling and curettage was carried out in that area for 4 weeks consecutively through scaling and curettage instead of going for a excision. The patient was simultaneously encouraged for regular brushing and flossing for 2 times a day for 4 weeks. After 4 weeks there was no growth visible clinically (Figure 5). It was totally eliminated. The patient was recalled every month for checkup and there was no recurrence even at the end of 6 months (Figure 6).

## 3. Conclusion

With the presentation of this paper it can be concluded that the combinations of various etiological factors might have caused the inflammatory tissue to cross the threshold from regular gingivitis to granuloma formation. The lesion was painless as nerves do not proliferate within the reactive hyperplastic tissue. It does not necessarily always require invasive excisional treatment; although surgery is successful in minimizing the recurrence of lesion, it often results in functional and esthetic impairment of the soft tissue morphology. So, the consideration should also be given to simpler and noninvasive treatment protocol procedures that resolve the lesion while preserving and improving the mucogingival complex.
